# Atherosclerotic-dose TMAO accentuates redox imbalances and motor dysfunctions in a MPTP mouse model of Parkinson’s disease

**DOI:** 10.3389/fnagi.2026.1871569

**Published:** 2026-07-29

**Authors:** Paul-Ştefan Panaitescu, Ioana Bâldea, Vlad Alexandru Toma, Alexandra-Maria Nuţu, Claudia-Andreea Moldoveanu, Alexandra Sevastre-Berghian, Ana-Maria Vlase, Irina Camelia Haranguş, Carmen Costache, Simona Clichici, Gabriela Adriana Filip

**Affiliations:** 1 Department of Microbiology, “Iuliu Hatieganu” University of Medicine and Pharmacy, Cluj-Napoca, Romania; 2 Department of Physiology, “Iuliu Hatieganu” University of Medicine and Pharmacy, Cluj-Napoca, Romania; 3 Department of Medical and Health Sciences Babes-Bolyai University, Cluj-Napoca, Romania; 4 Biological Research Institute, Cluj-Napoca, Romania; 5 Department of Molecular Biology and Biotechnology, Faculty of Biology and Geology, Babeş-Bolyai University, Cluj-Napoca, Romania; 6 Department of Pharmaceutical Botany, Faculty of Pharmacy, “Iuliu Haţieganu” University of Medicine and Pharmacy, Cluj-Napoca, Romania; 7 Department of Anatomy and Embriology, “Iuliu Haţieganu” University of Medicine and Pharmacy, Cluj-Napoca, Romania

**Keywords:** atherosclerotic, gut-brain axis, inflammation, MPTP, neurodegeneration, oxidative stress, Parkinson’s disease, TMAO

## Abstract

Parkinson’s Disease (PD) involves the loss of dopaminergic neurons and the formation of Lewy bodies consisting of alpha-synuclein (αSin) aggregates. Trimethylamine N-oxide (TMAO), a gut microbiota metabolite, has emerged as a molecule of interest due to pro-inflammatory, pro-oxidative, and neurodegenerative effects. We aim to assess whether lower doses of TMAO (40 mg/kg bw, gavage, once daily, day 0–42), as those used in atherosclerosis experimental models, can exacerbate oxidative stress, neuroinflammation, motor dysfunction, and neurodegeneration in a semi-acute 1-methyl-4-phenyl-1,2,3,6-tetrahydropyridine (MPTP) PD model (30 mg/kg bw MPTP, intraperitoneal, once daily, day 20–24). Motor behavioral testing functionally validated the model, with MPTP impairing performance across Rotarod latency time (*p* < 0.05), Wire Hang number of reaches and falls (*p* < 0.05), and Pole test time to descent (*p* < 0.01 MPTP vs. Control, *p* < 0.05 MPTP+TMAO vs. Control). A synergistic effect between TMAO and MPTP was observed in pole-turning time (*p* < 0.05). Increased TMAO serum was confirmed in treated animals (*p* < 0.05 TMAO vs. Control; *p* < 0.001 MPTP+TMAO vs. Control). Loss of tyrosine hydroxylase (TH) positive thalamic fibers, as well as substantia nigra neuronal fiber degeneration, and dopamine depletion was observed in the MPTP groups. A significant reduction in tyrosine hydroxylase (TH) expression in the midbrain was observed exclusively in the TMAO+MPTP group (*p* < 0.05 vs. Control). Neuroinflammatory profiling revealed selective elevation of IL-6 in the TMAO+MPTP group (*p* < 0.05), without microglial activation (*p* > 0.05), whereas NF-κB p65 was paradoxically increased only in the TMAO (*p* < 0.05) and MPTP (*p* < 0.05) groups. We hypothesize that this divergent pattern may indicate a shift toward necrotic rather than apoptotic cell death in the combined exposure group. MPTP and TMAO independently induced pro-oxidative states, evidenced by GSH depletion (*p* < 0.0001 MPTP vs. Control; *p* < 0.001 MPTP+TMAO vs. Control) and compensatory SOD upregulation (*p* < 0.01 TMAO vs. Control; *p* < 0.05 MPTP vs. Control and MPTP+TMAO vs. Control). The combination amplified the redox imbalance with significant elevation of MDA in the TMAO+MPTP group (*p* < 0.001 vs. Control and vs. TMAO; *p* < 0.01 vs. MPTP). These findings carry clinical relevance in the context of Parkinson’s disease, suggesting that gut microbiome-derived metabolites may represent a pathological connection capable of increasing both cardiovascular risk and the progression of neurodegenerative disorders.

## 1 Introduction

PD is a neurodegenerative disorder characterized by the loss of dopaminergic neurons and the formation of alpha-synuclein (αSyn) aggregates in the central nervous system (CNS), also known as Lewy bodies. The pathogenicity of the development of these characteristics is yet to be fully understood. This fact, as well as the high social and economic burden (Yang et al., 2020) associated with the disease, poses a challenging framework that implies the need for animal models in order to explore possible molecules and pathways involved in the development of the disease.

Up to now, two important factors in the development and progression of the disease have been identified—the immune pro-inflammatory response and oxidative damage (Mudimela et al., 2022; Schapira, 2008). The lesions are ultimately caused by oxidative damage due to oxidative imbalance triggered by excess oxygen reactive species (ROS), neuroinflammation, and energetic metabolism impairment (Dinan and Cryan, 2017). The gradual accumulation of ROS in neurons increases the secretion of cytokines, microglial activation, and apoptosis (Westfall et al., 2017). Possible exogenous sources of ROS include radiation, medicine, metabolites, and toxins (Mani, 2015). The endogenic ROS model is based on respiratory chain modulation in the mitochondria (Kim et al., 2015), with a reduction in complex I of the respiratory transport chain (Blesa et al., 2015). It is known that complex I inhibitors, such as MPTP, lead to cytotoxic effects on dopaminergic neurons—a reason for using MPTP to induce PD like in animal models (Lin and Beal, 2006). Gut health also seems to play an important role in the development and progression of PD (Sampson et al., 2016; Westfall et al., 2017). Braak et al propose the hypothesis of αSyn accumulation in the enteric nervous system that is afterwards advanced through the vagus nerve to the brain in a process comparable with the advancement of prions (Del Tredici and Braak, 2008). A more recent hypothesis proposes 2 pathogenic pathways, gut-first and brain-first, with the first being the one described by Braak and the second describing first the appearance of αSyn in the CNS and the progression to the enteric nervous system (ENS) through the vagus (Borghammer and Van Den Berge, 2019). These hypotheses are supported by the previous identification of αSyn in enteric nervous system, the vagus and glossopharyngeal nerves (Braak et al., 2003; Shannon et al., 2012). The injection of αSyn lysate obtained from a patient with PD in the intestinal wall of a rat leads to migration αSyn through the vagus nerve and subsequently, in connected regions of the brainstem (Holmqvist et al., 2014). Additionally, the vagotomy is associated with reduced odds for developing PD (Svensson et al., 2015).

Given the importance that gut health plays in the development of the disease, gut microbiota, gut-microbiota metabolites, and proinflammatory molecules are a central focus in unlocking the pathogenesis of PD. One of the most well-known gut metabolites, with demonstrated systemic proinflammatory effect, is trimethylamine N-oxide (TMAO) (Caradonna et al., 2025). Trimethylamine (TMA) results from the metabolism of choline and carnitine by the *Bacilota, Pseudomonadota, Actinobacteria, and Fusobacteria* phyla (Arias et al., 2020; Coutinho-Wolino et al., 2021; Kalnins et al., 2015; Martinez-del Campo et al., 2015; Rath et al., 2020). The enzyme cluster implicated in the production of TMA is the CutC/CutD gene cluster that can be found in bacterias that are normaly found in limited abundance in normal gut microbiota, such as *Klebsiella, Escherichia*, *Streptococcus* and *Enterobacter* (Craciun and Balskus, 2012). The resulting TMA is then absorbed and oxidized in the liver, by flavin-containing monooxygenase 3 (FMO3) leading to the production of TMAO (Bennett et al., 2013). Previously, this TMA/FMO3/TMAO pathway has also been linked with diabetes (Schugar et al., 2017), rasing another possibility of influence in PD and cardiovascular diseases through altered glucidic metabolism. The resulting TMAO is then predominantly eliminated in urine (Velasquez et al., 2016), although in cases of dysbiosis, TMAO can be reduced in the gut back to TMA (Fennema et al., 2016). Once in the brain, TMAO activates NF-κB, leading to microglial activation, astrocytic activation and NF-kB phosphorylation, leading to priming the NLRP3 inflammasome and cytokine gene expression (Mu and Wang, 2025). Cytokines, primarily IL-1β, IL-6 and TNFα, are both produced by and maintain microglial activation (Erdag and Haskologlu, 2025). Mice given dietary TMAO exhibit increased brain aging and cognitive impairment, most likely brought on by increased oxidative stress, mitochondrial dysfunction, and suppression of mTOR signaling in the brain (Li et al., 2018). Damaged mitochondria are both a *source* of ROS and an *activating signal* for the NLRP3 inflammasome, creating a feedforward loop (Prajapati et al., 2023). The buildup of ROS leads to increased malondialdehyde (MDA) levels. When combined with the previously mentioned effects, this leads to neuronal cell death, via apoptosis, pyroptosis, and necroptosis (Ji et al., 2022). MPTP is used as a model of PD based on toxicity, by directly inhibiting mitochondrial complex I, leading to NLRP3 inflammasome activation through mitochondrial ROS and microglial activation (Dou et al., 2018; Lee et al., 2019).

**FIGURE 1:**
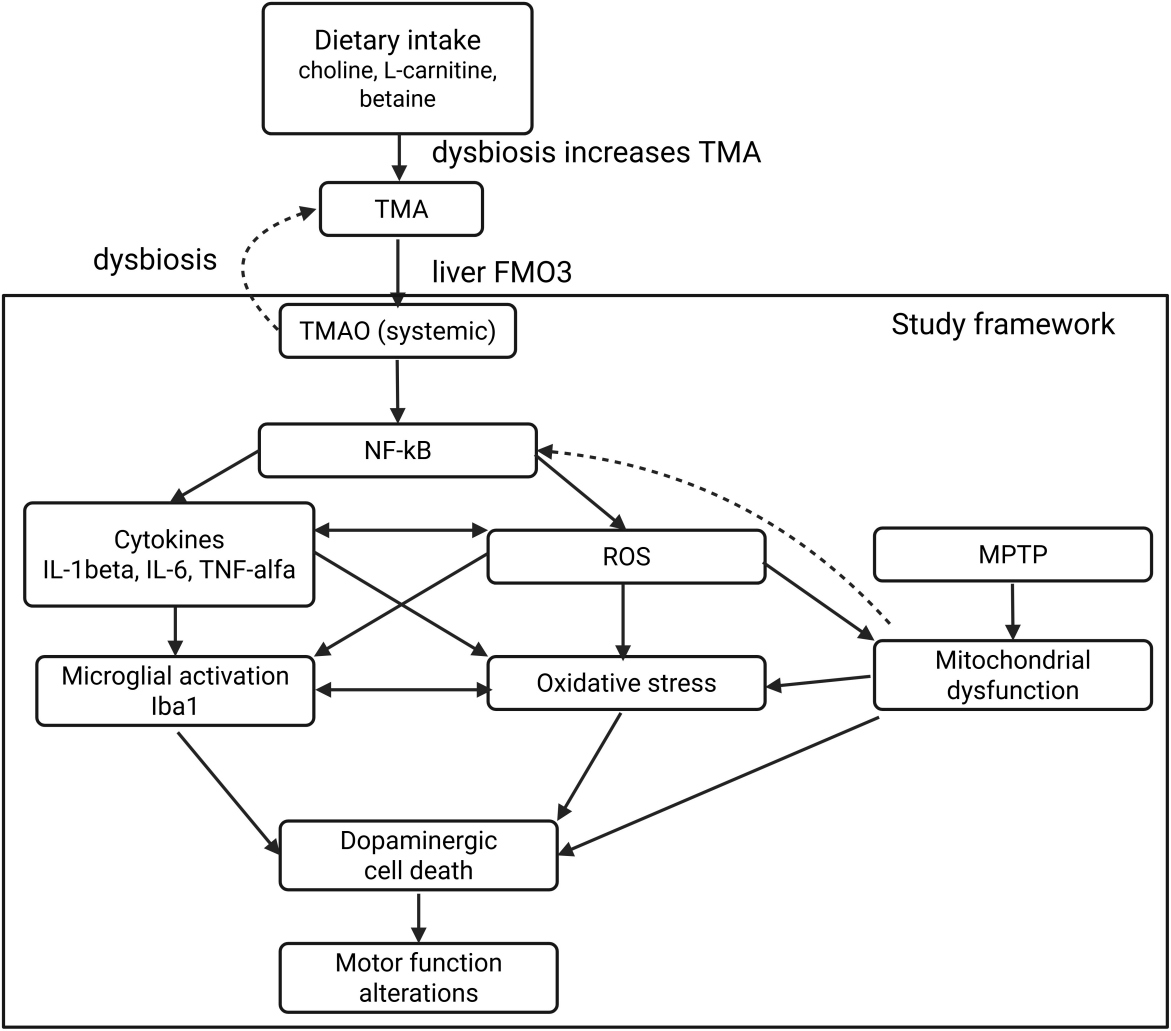
Experimental framework of the experiment. Created with BioRender.com.

Previous observations found that increased circulatory levels of TMAO were associated with inflammation, carbohydrate metabolism alteration, and neurodegeneration (Janeiro et al., 2018). An increase of circulatory and cerebrospinal TMAO is observed with aging, both in humans and in mice (Lee et al., 2022). A 2023 study showed that high doses of TMAO in the drinking water of mice challenged with MPTP increased neuroinflammation and motor dysfunction, and induced microglial and astroglial activation with high levels of TNF-alpha and IL-1 beta in the brain (Quan et al., 2023). Given the overlap between the PD model and TMAO downstream effects, as well as already established effects of high doses of TMAO in this model, we propose a further investigation to assess whether lower atherosclerotic doses (Florea et al., 2024) maintain the same effects, thus opening the discussion of the overlaps between cardiovascular risk and neurodegenerative diseases. Our study aims to evaluate the effects on redox imbalance, inflammation, and neurodegeneration in PD experimental model, the experimental framework being presented in Figure 1. This assessment is even more important because the previous studies already established that TMAO levels are increased in atherosclerotic and dementia patients (Gatarek et al., 2025; Heianza et al., 2017; Schiattarella et al., 2017; Yaqub et al., 2024; Zhu et al., 2020). While atherosclerosis is of high relevance to PD through a possible shared inflammatory pathway (Grosu et al., 2023), the pathology of the small vessels could be of higher interest, as cerebral small vessel disease could interact with the pathogenesis of PD (Visser et al., 2024). Moreover, TMAO and its metabolites are associated with the progression of cerebral small vessel disease (Chen et al., 2021).

## 2 Materials and methods

### 2.1 Animal grouping, disease induction, intervention, and timeline

**FIGURE 2:**
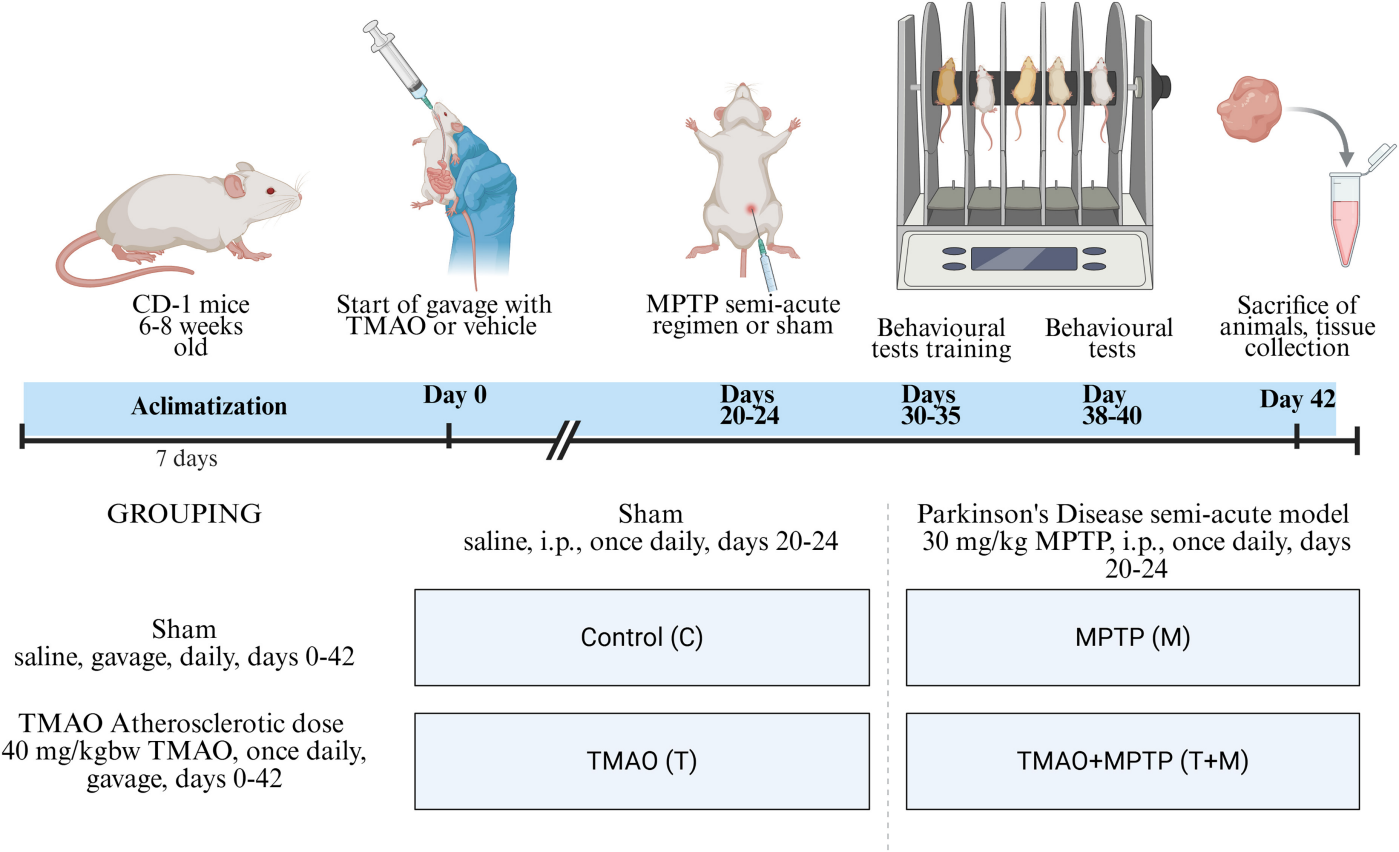
Timeline of the experiment. Acclimatization time—7 days; the intervention (days 0–42)—daily oral gavage with TMAO (40 mg/kg bw); MPTP semi-acute model induction—(days 20–24); Behavioral tests training (days 30–35) and trials (Florea et al., 2024; Heianza et al., 2017; Zhu et al., 2020). Sacrifice of animals, tissue collection—day 42. Grouping of animals in 4 groups (Control, MPTP, TMAO, MPTP+TMAO) through randomization. Created with BioRender.com.

A total of 46 CD-1 male mice, 6–8-week-old (29.33 g ± 1.14 g), were provided by the University of Medicine and Pharmacy “Iuliu Hatieganu” Cluj-Napoca Biobase. The animals were housed at constant temperature and humidity (24 ± 2 C, 55% ± 10%) with a 12 h day/night cycle. *Ad libitum* access to water and standard chow was provided. After transport, the animals were housed under the new conditions for 7 days before the start of the experiment. The timeline of all procedures is shown in Figure 2.

The animals were randomized in 4 experimental groups: Control group (*n* = 12), TMAO group (*n* = 12), MPTP group (n = 12), and MPTP+TMAO group (*n* = 12). The disease model was induced in two groups, MPTP and MPTP+TMAO, using a subacute model (30 mg/kg bw MPTP, intraperitoneal, days 20–24). The Control and TMAO groups received saline intraperitoneal, in the same volume and same posology. The TMAO and MPTP+TMAO group received the intervention—an atherosclerotic dose of TMAO (40 mg/kg bw, gavage) once daily, in the morning from days 0 to 42. The Control and MPTP groups received equivalent volumes of saline through gavage, once daily. Motor function was quantified using the following behavioral tests: Rotarod test, Wire Hang test, Pole test, OFT. Training was performed in days 30–35, and the trials were performed in days 38–40.

Animals were sacrificed through cervical dislocation on the 42nd day. The brains were processed in accordance with previously published protocols, depending on their purpose. For immunohistochemistry (*n* = 3/group), the brains were immediately placed in 10% neutral-buffered formalin. For western blot (*n* = 3/group), the brains were placed on ice, and the midbrain was dissected and stored at −80 °C for future analysis as per previously published protocol (Rodríguez-Cruz et al., 2019). For oxidative stress markers and TNF-α evaluation (*n* = 4–6/group), the brain was placed immediately on ice, the midbrain was dissected, and the samples were stored at −80 °C for future analysis. All animal work respected EU Directive 2010/63 on the protection of animals regarding the three Rs of ethical animal research used for scientific purposes, as well as the local legislative framework. This fact was validated by the approval of the Ethics Committee of the University of Medicine and Pharmacy “Iuliu Hatieganu” Cluj-Napoca (DEP119/11.04.2023) and The National Sanitary Veterinary and Food Safety Authority (A.N.S.V.S.A) with the project authorization number 381 from 25th August 2023.

### 2.2 Motor function tests

To evaluate the motor function of mice, behavioral training was performed for multiple days (days 30–35), followed by the trials (days 38–40). The rotarod test was performed in accordance with a previously published protocol (González-Cabrera et al., 2024) using a rotarod apparatus (Ugo Basil RotaRod, Gemonio, VA, Italy) for a total of three trials for each animal. The resulting data were weighted with body weight, as a previous study has shown that the test is influenced by the weight of the animal (Mao et al., 2015). Pole test was applied in accordance with previously published protocols, performing three trials per animal and quantifying the time it takes to turn on the pole (Time to turn), and the total time it takes to descend to the base of the pole (Time to descent) (Krout et al., 2024). The wire hang test was performed as previously published protocol, and the number of times the animal reaches the lateral poles (Number of reaches) as well as the number of times the mice fell from the wire (Number of falls) were quantified (Kam et al., 2024). Open field test (OFT) was used to evaluate general locomotion and exploratory behavior for mice (Ugo Basil Animal Mazes for Video-Tracking, Gemonio, VA, Italy). For each animal, the behavior was continuously recorded through a visual tracking system (Smart Basic Software version 3.0 Panlab Harvard Apparatus, Barcelona, Spain). The animals were freely allowed to explore an open field arena (50 × 50 × 40 cm) for 3 minutes (Sun et al., 2025). The total, peripheral, and central travelled distance, the number of entries in the center and periphery, as well as the center time ratio were analyzed.

### 2.3 TMAO quantification

In the final day of the experiment, retroorbital sinus blood was collected under mild anesthesia (2.5 mg/100 g bw ketamine 10% and 50 mg/100 g bw of xylazine hydroxichloride 2%). The blood was centrifuged for 10 min at 6500 rpm, and serum was immediately stored at -80 °C for future analysis. Serum TMAO levels were assessed through liquid chromatography–tandem mass spectroscopy (LC-MS/MS) (Agilent 1100 LC-MSD-Trap-SL, Agilent Technologies, Santa Clara, CA, United States) using a method already described (Le et al., 2019).

### 2.4 Oxidative stress parameters, cytokines, and dopamine levels

Oxidative stress parameters, reduced glutathione (GSH), oxidized glutathione (GSSG), GSH/GSSG ratio, and malondialdehyde (MDA) were evaluated from the midbrain tissue (*n* = 6/group). Homogenization of the brain tissue was performed on ice (POLYTRON PT 1200 E, Kinematica, Malters, Switzerland) in 50 mM TRIS and 10 mM EDTA buffer at pH 7.5. Resulting samples were centrifuged (1,000 × g, 10 min, 4 °C) and the supernatant collected and stored in pre-labeled sterile Eppendorf tubes, transported on crushed ice and immediately frozen at -80 °C until further analysis. Sample protein content was determined through the Bradford method (Bradford, 1976). MDA levels were quantified fluorometrically as end products of polyunsaturated fatty acid peroxidation, using the protocol established by Conti et al. (1991). GSH and GSSG levels were measured following a previously described method (Vats et al., 2008). For MDA, GSH, GSSG the results were expressed as nM/mg protein. TNF-α level was measured from the midbrain tissue homogenates (*n* = 6/group) using mice-specific ELISA kits, as per the manufacturer’s instructions (E-EL-M3063; Elabscience, Houston, TX, United States). Absorbance readings were performed at 450 nm, and the results were expressed as pg/mg protein. Dopamine levels were quantified using General Dopamine ELISA Kit (catalog no. E0851Ge, EIAAB Science, Wuhan, China), with results being expressed as ng/mg protein.

### 2.5 Western blot

The midbrain section (*n* = 3 per group) was dissected (Rodríguez-Cruz et al., 2019), and the expression of TH, Iba1, IL6, IL1-β, SOD, NF-κB, NF-κB p50, NF-κB p65, and Beta-actin was analyzed (*n* = 3). The tissue was lysed with an Igepal-nonidet 1% buffer (Sigma–Aldrich Chemicals GmbH, Munich, Germany), 1% protease inhibitor complex (Sigma–Aldrich Chemicals GmbH, Munich, Germany), and phosphate-buffered saline for 1 h on ice. The resulting lysates (20 μg protein/lane) were separated by electrophoresis on 8% SDS PAGE gels and subsequently transferred to polyvinylidenedifluoride membrane by using the Biorad Miniprotean system (BioRad, Hercules, California, United States). The blots were blocked using Starting BlockTM (TBS) Blocking buffer (Thermofisher Scientific, Rockford, United States) and then incubated with antibodies against tyrosine hydroxylase (TH) (E-AB-40585, Elabscience, Huston, United States), Iba1 (E-AB-64147, Elabscience, Huston, United States), IL-6 (C12-1-hIL-6, SCBT, Dallas, United States), IL-1β (pAB FNab04209, FineTest, Biolake, China), SOD (sc-17767, SCBT, Dallas, United States), NF-κB (FNab10334, FineTest, Biolake, China), NF-κB p50 (FNab10334, FineTest, Biolake, China), NF-κB p65 (sc-136548, SCBT, Dallas, United States) and Beta-actin (M1-114279, Antibodies online, Limerick, United States) diluted 1:500 and the corresponding secondary antibodies (np 7074S, Cell Signalling Technology, Danvers, United States and W4021, Promega, Madison, United States) at a dilution of 1:1,000. Supersignal West Femto Chemiluminis-cent substrate (Thermo Fisher Scientific, Rockford, United States) and a Gel Doc Imaging system equipped with a XRS camera and Quantity One analysis software (BioRad, Her-cules, CA, United States) were used to visualize and detect the target proteins. Beta-actin was used as a protein loading control.

### 2.6 Immunohistochemistry and histology

Brain samples (*n* = 3 per group) were collected for immunohistochemical (IHC) and histological analyses. Tissues were fixed in 5% neutral-buffered formalin and embedded in paraffin. Paraffin-embedded sections were processed for immunohistochemical detection of tyrosine hydroxylase (TH) using a horseradish peroxidase (HRP)-based system with 3,3’-diaminobenzidine (DAB) as chromogen, as well as for Bielschowsky silver staining to visualize neuronal structures. The sections were deparaffinized in three consecutive xylene baths, 5 min each, followed by rehydration through a graded ethanol series (100, 96, and 70%; 5 min each), and rinsed in distilled water for 5 min. For TH staining, antigen retrieval was performed in 10 mM citrate buffer (pH 6.0) by heating at near-boiling temperature for 10 min, followed by cooling at room temperature for 30 min. Sections were washed in distilled water and Tris-buffered saline (TBS). Endogenous peroxidase activity was quenched using 3% hydrogen peroxide for 10 min at room temperature. After washing in TBS containing 0.1% Tween-20 (TBS-T), non-specific binding was blocked by incubating sections in 3% bovine serum albumin (BSA) for 1 h at room temperature. Sections were incubated with a primary anti-TH antibody (Sigma-Aldrich, MAB318) diluted 50x in TBS containing 1% serum and 0.1% Tween-20 overnight at 4°C. Following incubation, slides were washed in TBS-T (3 × 5 min). Detection was carried out using an HRP-conjugated secondary system (SignalStain^®^ Boost Detection Reagent, Cell Signaling Technology) for 30 min at room temperature. Immunoreactivity was visualized using a DAB substrate solution (SignalStain^®^ DAB Substrate Kit) for 10 min, and the reaction was stopped by rinsing in distilled water. Sections were counterstained with hematoxylin for 1 min and rinsed in water.

For Bielschowsky silver staining, a 10% silver nitrate (AgNO_3_) solution was prepared by dissolving 5 g AgNO_3_ in 50 mL distilled water. The stock developing solution consisted of 20 mL formaldehyde (37–40%), 0.5 g citric acid, and 2 drops of concentrated nitric acid, adjusted to 50 mL with distilled water. The working solution was freshly prepared by adding 20 drops of stock solution and 8 drops of concentrated ammonium hydroxide to 50 mL distilled water. Sections were incubated in 10% AgNO_3_ at 40°C for 15 min and rinsed three times in distilled water. Ammonium hydroxide was added dropwise to the silver solution until a clear ammoniacal silver complex (Tollens-like reaction) was obtained. Sections were then incubated in this solution at 40°C for 30 min until visible darkening occurred. Slides were subsequently transferred to the developing solution for approximately 1 min until a brown reaction product developed. To enhance staining intensity, 2 drops of 37% formaldehyde were applied directly onto each section prior to development. Following staining, sections were dehydrated through graded ethanol, cleared in xylene, and covers using a permanent synthetic mounting medium.

### 2.7 Statistical analysis

Statistical analysis was performed in GraphPad Prism version 9.3.0 on a Microsoft Windows 11 computer (GraphPad Software, San Diego, CA, United States).^1^ Data normality was assessed using the Kolmogorov–Smirnov and Shapiro–Wilk tests. When normality conditions were met, group differences were evaluated by one-way ANOVA, with post hoc comparisons being performed using Tukey’s test with Bonferroni adjustment for multiple comparisons. All data showed normal distribution, and as such, no non-parametric alternative was necessary. Unless stated otherwise, data are presented as mean ± SD with statistical significance set at *p* < 0.05.

## 3 Results

### 3.1 Motor function

**FIGURE 3:**
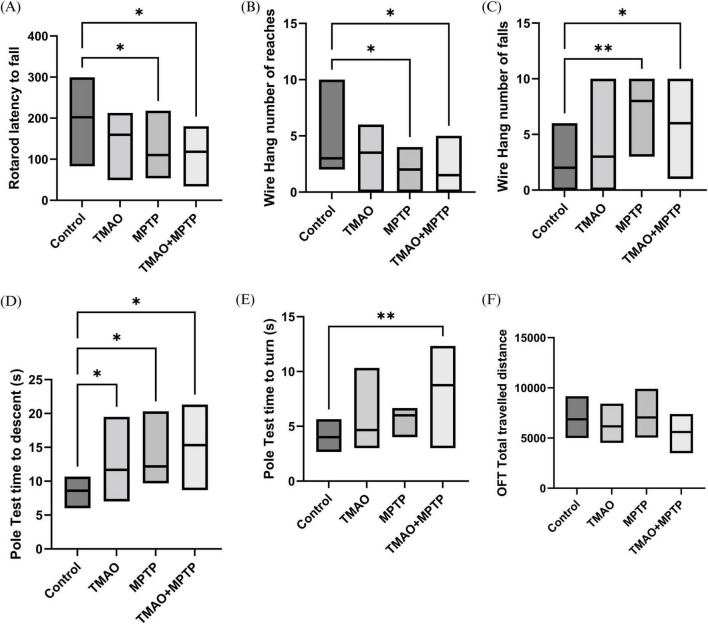
Motor function tests. **(A)** Rotarod—latency to fall, both MPTP (*p* < 0.05) and MPTP+TMAO (*p* < 0.05) groups show lowered latency to fall vs. Control. **(B)** Wire hang test—number of reaches is reduced by MPTP both in the MPTP (*p* < 0.05) and the MPTP+TMAO (*p* < 0.05) group, compared to the Control. **(C)** Wire hang—number of falls is increased in the MPTP (*p* < 0.01) and MPTP+TMAO group (*p* < 0.05) vs. Control. **(D)** Pole test—time to descent is increased in all groups vs. control (*p* < 0.05). **(E)** Pole test—time to turn is increased only in the MPTP+TMAO group vs. Control (*p* < 0.05). **(F)** Open field test—total travelled distance, no statistically significant differences were found. Results were expressed as mean ± SD, *p < 0.05, **p < 0.01. Created with BioRender.com.

In the rotarod test, MPTP decreased the latency time in both the group with treatment (*p* < 0.05) and without treatment (*p* < 0.05) (Figure 3A). The same findings can be observed in the wire hang test: MPTP lowered the reaches both in the group with TMAO (*p* < 0.05) and without TMAO (*p* < 0.05), as well as increased the number of falls, both in the MPTP group (*p* < 0.01), as well as the TMAO+MPTP group (*p* < 0.05) (Figures 3B,C). These findings functionally correlate with the lesions in the midbrain noticed microscopically. Regarding the effect of the TMAO, in the pole test, the administration of TMAO increased the time to descend (*p* < 0.05), while MPTP led to increased time to descent both in the group with TMAO (*p* < 0.05) and without TMAO (*p* < 0.05). No synergic effect was observed between TMAO and MPTP administration for time to descent (Figure 3D). Nonetheless, a synergic effect is observable in the time to turn on the pole, with only the TMAO+MPTP group having an increase of this parameter (*p* < 0.01) (Figure 3E). No significant statistical differences were observed in the parameters measured in OFT (Figure 3F).

### 3.2 TMAO quantification

**FIGURE 4:**
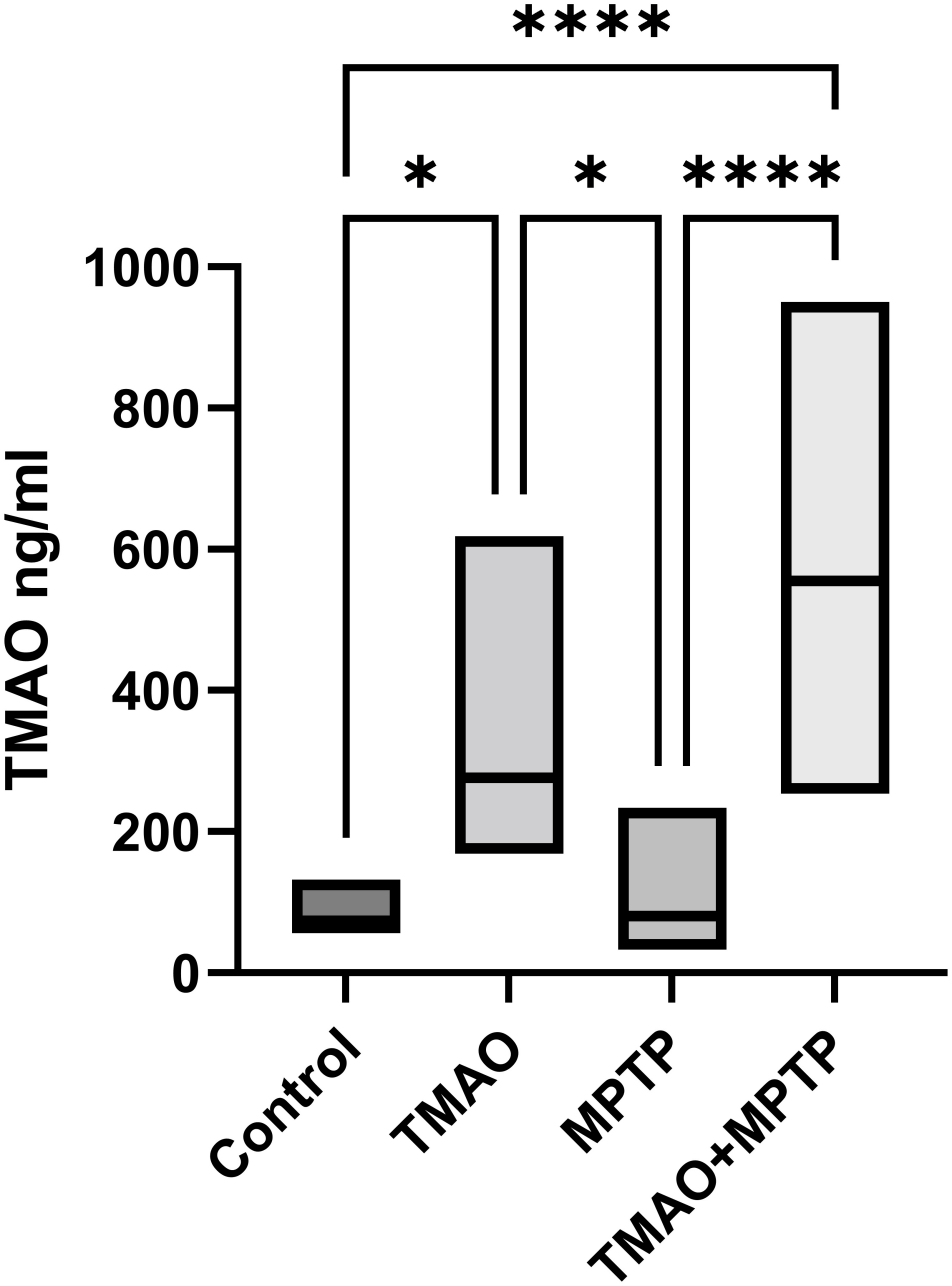
Serum levels of TMAO per experimental groups. TMAO serum concentration was elevated in the TMAO group vs. Control (*p* < 0.05) and vs. MPTP (*p* < 0.05). MPTP+TMAO group showed increased levels vs. Control (*p* < 0.0001) and vs. MPTP group (*p* < 0.0001). Results were expressed as mean ± SD, *p < 0.05, ****p < 0.0001. Created with BioRender.com.

LS-MS serum analysis shows statistically elevated TMAO concentrations following the administration of atherosclerotic daily doses of TMAO both in the TMAO group (*p* < 0.05) and the MPTP+TMAO group (*p* < 0.0001), compared to the control, as well as the MPTP group (Figure 4).

### 3.3 Oxidative stress markers

**FIGURE 5:**
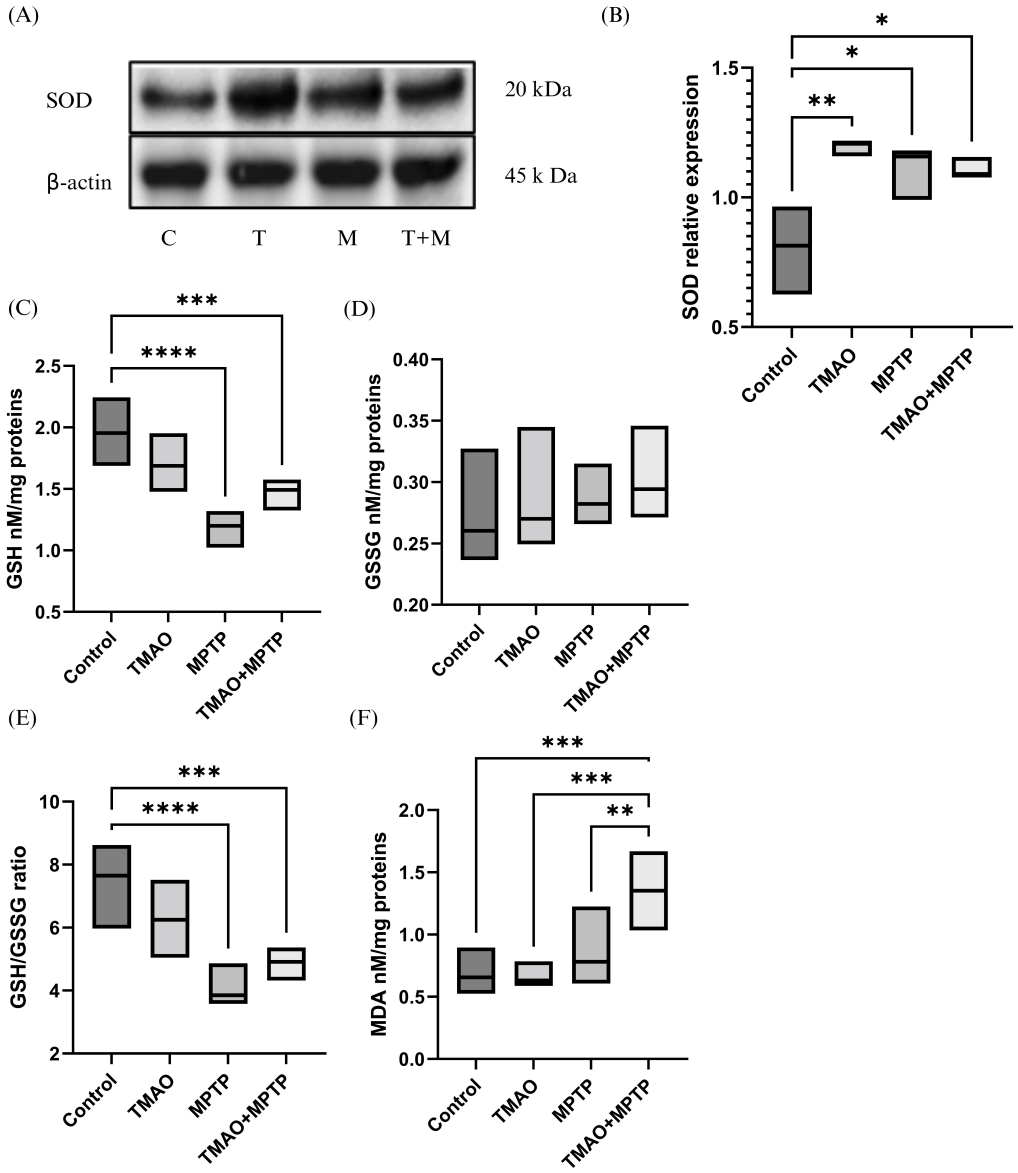
Oxidative stress parameters in midbrain of animals with experimental PD induced by MPTP. **(A,B)** SOD expression in the midbrain evaluated by western blot, showing increased levels in TMAO vs. Control (*p* < 0.01), MPTP vs. Control (*p* < 0.05) and MPTP+TMAO vs. Control (*p* < 0.05) Image analysis of western blot bands was done by densitometry; results were normalized to β-actin. **(C)** GSH levels in the midbrain, colorimetric assay, was lowered by MPTP, both in the MPTP (*p* < 0.0001) and the MPTPT+TMAO (*p* < 0.001) groups. **(D)** GSSG levels in the midbrain, colorimetric assay, showed no statistical differences. **(E)** GSH/GSSG ratio in midbrain, showed the same significant differences as the GSH levels. **(F)** MDA levels in the midbrain by fluorimetry, MPTP+TMAO induced lipid peroxidation compared to Control (*p* < 0.001), TMAO (*p* < 0.001) and MPTP (*p* < 0.001) groups. Results were expressed as mean ± SD, *p < 0.05, **p < 0.01, ***p < 0.001, ****p < 0.0001. Created with BioRender.com.

The protective anti-oxidant SOD activity was elevated as a response to TMAO (*p* < 0.01), as well as MPTP, both in the group without TMAO administration (*p* < 0.05) as well as the group treated with TMAO (*p* < 0.05) (Figures 5A,B). When it came to the glutathione reduced/oxidized balance, MPTP lowered GSH concentrations both in MPTP group (*p* < 0.0001) and the MPTP+TMAO group (*p* < 0.001) vs. Control, while not affecting the GSSG levels (Figures 5C,D). The same findings were also found in the case of the GSH/GSSG ratio, with MPTP lowering it both in the group without the intervention (TMAO) (*p* < 0.0001) and the group with the intervention (*p* < 0.001) (Figure 5E) Regarding MDA, only TMAO+MPTP showed increased levels when compared to the control (*p* < 0.001), TMAO (*p* < 0.001), and MPTP groups (*p* < 0.01), presenting a potential synergistic effect of the interaction and the disease model (Figure 5F). These results demonstrated that MPTP induced a pro-oxidative status with increased expression of SOD and depletion of GSH as a response, managing to prevent the peroxidation of lipids. In TMAO group, the pro-oxidative effect leads to the increased expression of SOD, in order to prevent lipid peroxidation and depletion of GSH. In combined regimen, a synergic effect was noticed on lipid peroxidation.

### 3.4 Neurodegeneration quantification by western blot, IHC, histology, and dopamine levels

**FIGURE 6:**
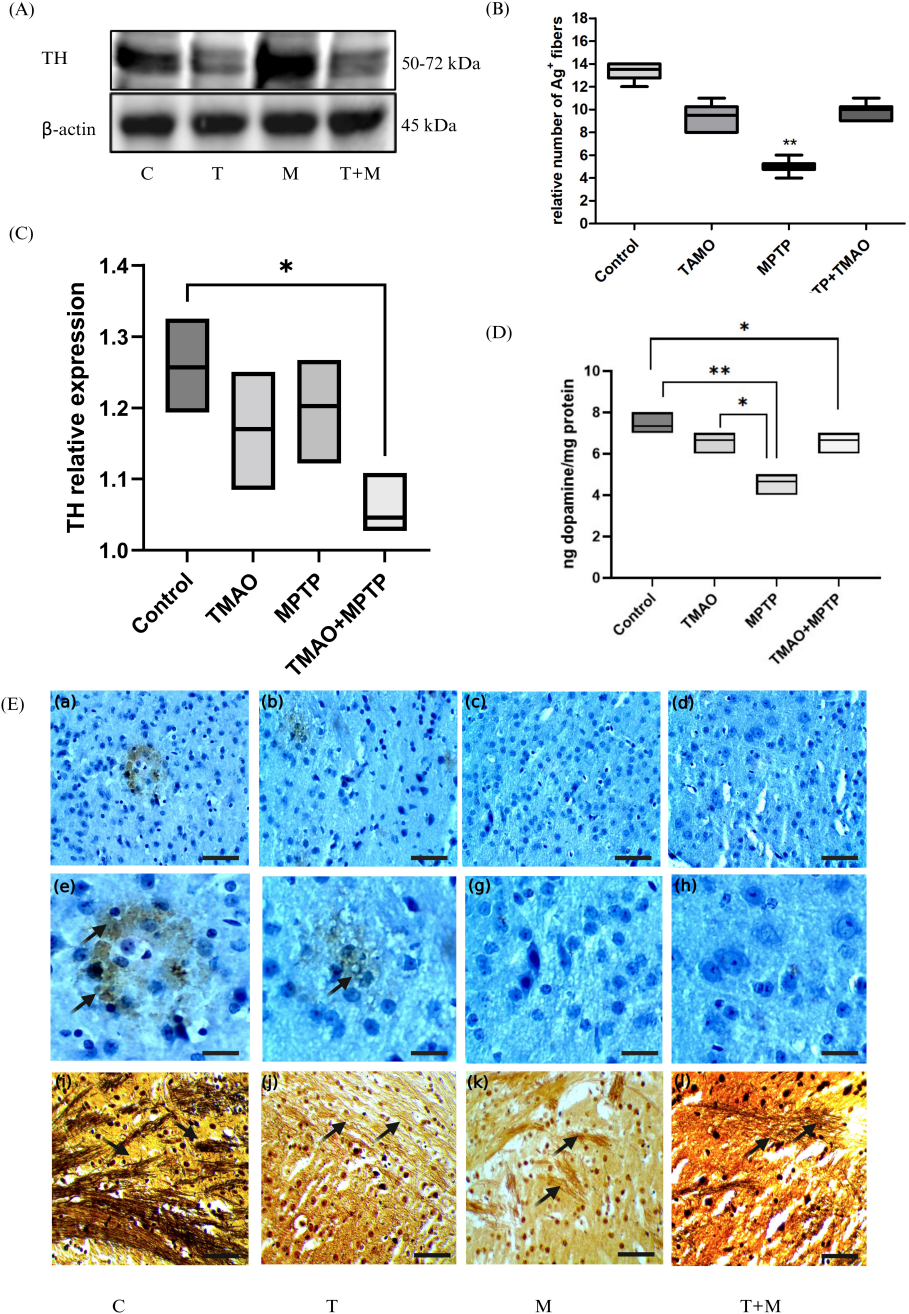
Neurodegeneration and dopaminergic cell loss were assessed by western blot, immunohistochemistry and histology. **(A,C)** Western blot analysis of TH protein expression in midbrain tissue showing statistically significant reduction in TH expression in the MPTP+TMAO group vs. Control (*p* < 0.05). Image analysis of western blot bands was done by densitometry; results were normalized to β-actin. **(B)** Quantification of the relative number of silver-impregnated fiber bundles per microscopic field area in the Control and experimental groups. The evaluation was performed by analyzing five different microscopic fields. Data are expressed as mean ± SD. Statistical analysis revealed a significant decrease in silver-impregnated material (**p < 0.01) in MPTP-exposed animals. **(D)** Dopamine concentrations. As compared to Control, after MPTP exposure, dopamine levels were significantly decreased (**P < 0.01), as well as after MPTP+TMAO combined treatment (*P < 0.05). In turn, TMAO exposure alone did not induce a significant reduction compared to the Control group, with dopamine levels remaining significantly higher than those observed in the MPTP-lesioned group (*P < 0.05). **(E)** Immunohistochemistry and silver staining panel (a-d) Representative TH IHC images in the thalamus acquired at 20 × magnification (scale bar: 50 μm) showing (a) Control, (b) TMAO, (c) MPTP, and (d) MPTP+TMAO groups. (e-h) Higher magnification images (40×; scale bar: 20 μm) of TH in thalamus: (e) Control, (f) TMAO, (g) MPTPT, and (h) MPTP+TMAO. Arrows indicate TH-positive cells. (i–l) Representative Bielschowsky silver staining of the substantia nigra (SN) at 20 × magnification (scale bar: 50 μm) in (i) Control, (j) TMAO, (k) MPTPT, and (l) MPTP+TMAO groups, highlighting neuronal fibers and neurodegenerative changes. Results were expressed as mean ± SD, *p < 0.05, **p < 0.01. Created with BioRender.com.

The absence of TH^+^ thalamic fibers in MPTP and TMAO+MPTP groups can be observed, with decreased expression in the TMAO group, and focal fibers in the Control group (Figures 6Ea–h). Morphological abnormalities, such as neuronal degeneration and increased Virchow-Robin space, can be noticed in the TMAO+MPTP group, with dark neurons present in the TMAO group (Figures 6Ea–h). In MPTP and TMAO+MPTP groups, nervous fiber degeneration can be noticed in substantia nigra (Figures 6Ef–l). Limited neurodegenerative effect was found through midbrain Western Blot, as only the TMAO+MPTP group showed a statistically significant reduction of tyrosine hydroxylase (TH) relative to the controls (*p* < 0.05), with no statistical difference for the MPTP group (Figures 6A,C). Under the action of MPTP, the density and thickness of silver-impregnated fibers decreased (Figure 6K) compared to the control group. In the MPTP group, this effect was more pronounced, but it was also observed in the MPTP+TMAO group, albeit to a lesser extent (Figure 6B). Both groups show comparable dopamine depletion (Figure 6D). These changes– demonstrate the pharmacologically induced lesion caused by MPTP, as well as the combined effect of TMAO and MPTP on the depletion of the TH-positive immunohistochemical response.

### 3.5 Neuroinflammation assessment in the midbrain of animals with experimental PD

**FIGURE 7:**
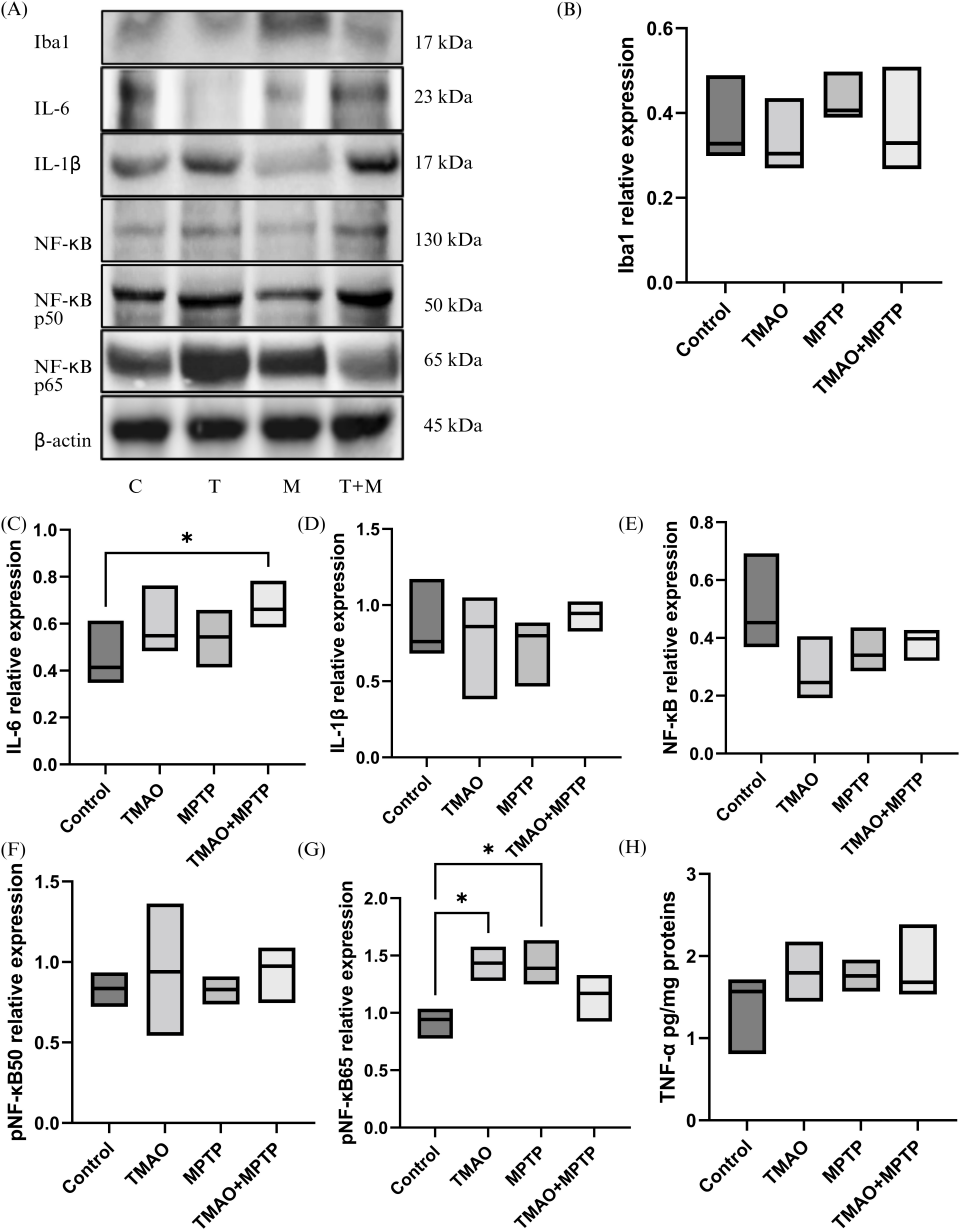
The parameters of neuroinflammation in midbrain of animals with experimental PD. **(A–G)** The expression of Iba1, IL-6, IL-1β, NF-kBp50, NF-kBp65, NF-kB in midbrain analyzed by western blot. Image analysis of western blot bands was done by densitometry; results were normalized to β-actin. **(H)** TNFalpha levels evaluated by ELISA (pg/mg P). TMAO+ MPTP group shows increased IL-6 expression compared to the control (*p* < 0.05), while NF-κB p65 was improved only in the TMAO and MPTP groups. Notably not the same effect was observed in the combined exposure. Results were expressed as mean ± SD. *p < 0.05. Created with BioRender.com.

No microglial activation was found, with Iba1 not being significantly altered between groups in the midbrain (Figures 7A,B). Interestingly, there is a significant increase in IL6 levels in the midbrain but only in the TMAO+MPTP group (*p* < 0.05), showing a possible synergic effect of TMAO and MPTP, neither producing effects on their own (Figure 7C). This change was not also observed in IL-1β levels, as no significantly statistical differences was observed between groups (Figure 7D). Regarding NF-κB and its phosphorylated subunits, only NF-κB p65 was altered, with TMAO (*p* < 0.05) and MPTP groups (*p* < 0.05) showing increased levels in the midbrain compared to the control (Figures 8E–G). The effect is absent in the TMAO+MPTP group (Figures 7E–G), while IL-6 is increased only in this group (Figure 7C). No statistical difference was observed between groups for TNFα (Figure 7H).

## 4 Discussion

### 4.1 PD model induction

In the present model, we aimed, through the administration of MPTP, to establish an experimental model of PD in order to investigate whether the association of TMAO amplifies the degenerative changes, inflammation, and oxidative stress associated with the disease. MPTP model offers the advantage of dopaminergic cell loss in the mouse midbrain that exhibits a topographic distribution comparable to that observed in human PD, with neurodegeneration predominantly affecting the ventral tier and lateral SNpc neurons, as well as posterior regions, while relatively sparing the more anterior and medial cell populations (Meredith and Rademacher, 2011). Nonetheless, this model also has some limitations, such as the absence of Lewy body inclusions (Meredith et al., 2004) and possible reversibility of the model since the TH reduction might also be due to the temporary suppression of TH expression MPTP-induced (Xu et al., 2005) Functionally, our model was validated by leading to impaired motor function—reduced latency in rotarod, increased time to descent in the pole test, increased number of falls, and lowered number of reaches in the wire hang test. Our findings show MPTP-induced nervous fiber destruction in the substantia nigra, accompanied by thalamic TH+ fiber loss. This loss observed in our model is consistent with previously described MPTP-induced neurodegeneration beyond the nigrostriatal system, with studies proving degeneration in the thalamus both in monkeys and mice MPTP models (Freyaldenhoven et al., 1997; Monje et al., 2020). Notably, the reduction in TH protein expression detected by western blot in the midbrain, as well as morphological abnormalities, was exclusive to the TMAO+MPTP group, suggesting a potentiating interaction between TMAO and MPTP. The limited reduction observed in western blot is in line with previous studies performed on CD-1 mice using MPTP, with results varying from no effect of the disease model (Rodríguez-Cruz et al., 2019) to 15% reduction in the acute model (Vidyadhara et al., 2019). Both groups show comparable dopamine depletion coupled with fiber loss. The additional TH suppression and MDA increase in MPTP+TMAO indicate TMAO adds an oxidative burden, without further reducing total tissue dopamine—possibly because turnover and release compensate for reduced synthetic capacity. Further studies that analyze the turnover ratio data (DOPAC/DA, HVA/DA) are needed in order to confirm this.

The observed neurological damage is downstream of the increase in redox imbalances found in MPTP-challenged groups and the inflammatory response. The limited inflammatory response could be due to the lower aggressiveness of the semi-acute model or the long period between disease induction and tissue sampling. A limitation in inducing the PD model is that CD-1 mice are known to exhibit lower susceptibility to MPTP than C57BL/6 mice due to having a larger number of TH-positive neurons and, as such, an increased dopaminergic reserve (Muthane et al., 1994). Nonetheless, there are also advantages in this strain—the results could translate to a larger human population since it is not inbred, such as C57BL/6, with a particular MPTP sensibility.

### 4.2 Effect of atherosclerotic dose TMAO

The administration of lower doses of TMAO induced its accumulation in the serum, despite the rapid urinary clearance of the molecule (Velasquez et al., 2016). The level of accumulation of TMAO is comparable to that described in diabetic mice, but reduced when compared to the choline chow (Kong et al., 2024). In comparison with the other studies using high exposure to TMAO in drinking water (Quan et al., 2023), we did not identify microglial activation, and only a limited increase in midbrain IL6 in the TMAO+MPTP group was identified. Quan et al.)2023) also found increases in TNFα, IL-1β, and IL-6 in the hippocampus produced by TMAO. As previously stated, TMAO is predominantly eliminated in urine (Velasquez et al., 2016), but in cases of gut-microbiota alterations TMAO can be reduced back to TMA (Fennema et al., 2016). This happens when bacteria that can use TMAO as terminal acceptors for anaerobic respiration through TMAO reductase systems, such as *Enterobacteriaceae*, become more abundant (Lock et al., 2022). This poses the possibility of a positive feedback loop that might ensure long-lasting dysbiosis if we have the blooming of an ecological niche that possesses both the CutC/CutD gene cluster, as well as TMAO reductase systems. As such exogenous TMAO administration might act as a dysbiosis factor, besides the pro-inflammatory factors. Also, once developed such a niche, it might lead to the reduction of bioavailability of choline, L-carnitine and betaine. Future research should expand upon our findings and search in which ecological configurations the gut microbiota might lead to comparable sustained TMAO levels.

Particular to our findings is the fact that NF-κB p65 expression was increased only in TMAO and MPTP groups, but not in the combined TMAO+MPTP group. Under physiological conditions, NF-κB—a heterodimeric transcription factor comprising p50 and p65 subunits—remains sequestered in the cytoplasm through its interaction with the inhibitory protein IκB (Aoki et al., 2009). Upon activation, NF-κB dissociates allowing the p50-p65 heterodimer to translocate to the nucleus, where it binds to specific cognate DNA sequences to regulate gene transcription (Thanos and Maniatis, 1995). Notably in our study, we observe a lack of increase in NF-κB p65 expression combined with no effect on IL-1β and the increase of IL-6 in the TMAO+MPTP group. The response was present in the TMAO and MPTP groups. When considered alongside the elevated IL-6 levels that is also only present in the TMAO+MPTP group, these findings may suggest a shift toward necrotic rather than apoptotic cell death. Recent studies have shown that TMAO can also lead to a panoptosis process that includes apoptosis, necroptosis and pyroptosis both in hippocampal neurons (Wang et al., 2025) and kidney cells (Shao et al., 2025). Our results raise the question if necroptosis could happen without a significant increase in TNF-α, the main mediator of the pathway (Koehler et al., 2024). We hypothesize that this could happen in the context of danger-associated molecular patterns (DAMP) (Kang et al., 2022), as TMAO has been proposed as a conditional DAMP (Sun et al., 2018). It is also established that the necroptosis process is governed by oxidative mechanisms, particularly through mitochondrial reactive oxygen species-driven lipid peroxidation (Zheng et al., 2024). NF-κB p65 is one of the main molecular switches that prevents necroptosis (Thapa et al., 2011), and in our case was decreased in the TMAO+MPTP group. Our findings point toward a possible overwhelming of the compensatory mechanisms that prevent necroptosis, but further studies should be performed to confirm our findings. Further *in vivo* studies should be performed to confirm this hypothesis by analyzing all the steps of our proposed mechanistic explanation. Further *in vitro* studies should be performed to assess whether there is a dose-dependent interaction between TMAO and MPTP.

Regarding the oxidative stress, glutathione results show a paradoxical finding as there is no relation between GSH and GSSG as it would be expected. This could be explained by multiple mechanism that MPTP induces. In the context of MPTP-induced dopaminergic neurodegeneration, GSH loss may also partly reflect overall neuronal loss rather than exclusively biochemical oxidation, further dissociating it from GSSG dynamics (Smeyne and Smeyne, 2013).

Morphological abnormalities were also found in the TMAO-treated groups, with dark neurons being observed in the TMAO group and increased neurodegeneration and Virchow-Robin space in the MPTP+TMAO group. The increase of the Virchow-Robin space could be highly relevant since it is considered a marker of small vessel disease (Rouhl et al., 2008). These alterations point toward the increased stress that circulating TMAO leads to in the brain. Also, only in the TMAO+MPTP group, a statistically significant reduction in TH expression in the midbrain was identified demonstrating the combined effect of MPTP and TMAO. Functionally, these findings are validated by the effect on the time to turn in the pole test produced by the combined regimen. This effect did not carry through to the descent time, as descent time was increased in all other groups when compared to the control. The pole test is mainly used to assess bradykinesia (Balkaya et al., 2013), but an increased time to turn might also point toward another symptom of PD, difficulty in initiating movement—akinesia (Sheta et al., 2024). This means that both TMAO and MPTP lead to bradykinesia, while their combination also produces akinesia. Our findings raise the question as to why the dopamine levels were not significantly lowered in the MPTP+TMAO group when compared to the MPTP group, which did not show the same sequence of results. We have established that both MPTP groups show fiber destruction and reduced dopamine vs. control, proving the disease model, fact functionally validated by the Rotarod, wire hang and pole test—time to descent. Also, we have found that MPTP+TMAO significantly reduces TH and increases MDA on top of this shared lesion, with downstream motor effects on the capacity to turn on the pole of the mice, which can be interpreted as akinesia. As to why the dopamine and TH relationship differs between groups, this points toward compensatory effects, dopamine turnover or cell death pathways that require further data in order to establish.

### 4.3 Limitations

Regarding limitations, our study lacks the full coverage of the mechanistic framework in which TMAO and MPTP interplay in this Parkinson’s Disease model. This means a lack of clarity toward the meaning of certain results and forcing us to only hypothesize a possible shift toward necroptosis that might have happened in the TMAO+MPTP group. Non-statistically significant results for microglial activation also stunt our findings, not being in line with previously published findings. This might be related to the longer period of time from disease induction to sample collection. Further research should clarify the time and dose-dependent evolution of the microglial activation in PD models. The MPTP model itself also poses a limitation, as it is still debated whether it induces consistent dopaminergic cell loss or if it only leads to a transitory downregulation of TH, without permanent damage to the dopaminergic neurons.

### 4.4 Clinical implications

PD is a complex neurodegenerative disorder in which vascular comorbidities frequently coexist, with cerebral small vascular disease being of interest due to potential interactions (Visser et al., 2024). Microbiome-derived metabolites such as TMAO may represent a pathological link capable of stimulating the cardiovascular risk and accelerating the progression of neurodegenerative pathology (Ji et al., 2022). Elevated TMAO levels in blood have been associated with endothelial dysfunction, pro-inflammatory macrophage polarization, impaired reverse cholesterol transport, and platelet hyperreactivity, each of which contributes independently to the heightened cerebrovascular burden noticed in Parkinson’s patients (Gatarek et al., 2025; Heianza et al., 2017; Schiattarella et al., 2017; Wang et al., 2021; Yaqub et al., 2024; Zhu et al., 2020). Beyond the vascular effects, emerging evidence suggests that TMAO induces redox imbalances, aggravates mitochondrial dysfunction, and potentiates alpha-synuclein aggregation, thereby establishing a mechanistic framework through which gut dysbiosis can accelerate central nervous system degeneration (Chen et al., 2020; Qiao et al., 2023; Quan et al., 2023). Our findings prove that a limited dose of TMAO can also lead to accumulation in the blood and create a redox imbalance environment with consequences on motor function, neuroinflammation, and neurodegeneration. The role that TMAO also plays in the pathogenesis of cardiovascular disease poses a question of the possible overlap of disease progression with neurodegenerative diseases. Further research is needed to clarify if circulating TMAO could be a marker for disease progression for both and whether interventions in limiting its bioaccumulation might lead to disease-modifying effects.

## 5 Conclusion

This study demonstrates that TMAO potentiates the neurodegenerative, neuroinflammatory, and oxidative effects of MPTP in a mouse model of Parkinson’s disease. While MPTP alone reliably induced motor deficits, dopaminergic fiber loss in the substantia nigra and thalamus, dopamine depletion, GSH depletion, and compensatory SOD upregulation, the combined TMAO+MPTP regimen produced more severe outcomes across multiple domains.

Synergistic effects were most evident in lipid peroxidation (elevated MDA), midbrain TH protein reduction, IL-6 upregulation, and akinesia on the pole test—none of which reached significance under either treatment alone. Notably, NF-κB p65 was increased in the TMAO and MPTP groups independently but not in the combined group, suggesting that this protective anti-necroptotic mechanism may be overwhelmed under dual exposure, requiring further studies to explore this possibility.

At an atherosclerotic dose, TMAO alone also produced oxidative stress and morphological brain changes, including dark neurons and enlarged Virchow-Robin spaces, indicating direct neurotoxic potential independent of MPTP. Taken together, these results suggest that chronically elevated circulating TMAO may represent a modifiable environmental risk factor capable of amplifying neurodegeneration in the context of PD, warranting further mechanistic and dose-response investigation. These observations highlighted the gut-brain axis relevance, especially TMAO in PD, as a potential therapeutic and biomarker target, justifying further investigation for dietary modification, microbial-targeted interventions (Kalagi et al., 2019; Panaitescu et al., 2024), or direct pharmacological inhibition of TMAO biosynthesis (Kalagi et al., 2019) for disease modification.

## Data Availability

The original contributions presented in the study are included in the article/Supplementary material, further inquiries can be directed to the corresponding authors.

## Footnotes

1
https://www.graphpad.com/
