# Supplementary material for: Atherosclerotic-dose TMAO accentuates redox imbalances and motor dysfunctions in a MPTP mouse model of Parkinson’s disease
Source: Front Aging Neurosci. 2026 Jul 29;18:1871569. doi: 10.3389/fnagi.2026.1871569 (PMC13463374; doi:10.3389/fnagi.2026.1871569)
Supplement: Supplementary file 1 [file Data_Sheet_1.docx]

TH expression in the Midbrain

15 kDa

20 kDa

250 kDa

C	T	M	M+T

C	T	M	M+T

C	T	M	M+T

25 kDa

37 kDa

100 kDa

150 kDa

50 kDa

75 kDa

Supplementary figure 1. Full uncropped Blots image(s) for TH (50-72 kDa)

C	T	M	M+T

C	T	M	M+T

C	T	M	M+T

15 kDa

20 kDa

25 kDa

37 kDa

50 kDa

75 kDa

100 kDa

150 kDa

250 kDa

Supplementary figure 2. Full uncropped Blots image(s) for Iba1 (17 kDa)

C	T	M	M+T

C	T	M M+T

C	T	M	M+T

C	T	M	M+T

250 kDa

250 kDa

20 kDa

25 kDa

37 kDa

50 kDa

75 kDa

100 kDa

150 kDa

150 kDa

15 kDa

20 kDa

25 kDa

37 kDa

50 kDa

75 kDa

100 kDa

Supplementary figure 3. Full uncropped Blots image(s) for IL-6 (23 kDa)

C	T	M	M+T

C	T	M	M+T

C	T	M	M+T

20 kDa

25 kDa

15 kDa

10 kDa

37 kDa

50 kDa

75 kDa

150 kDa

100 kDa

250 kDa

Supplementary figure 4. Full uncropped Blots image(s) for IL-1β (17 kDa)

10 kDa

250 kDa

15 kDa

20 kDa

25 kDa

37 kDa

50 kDa

75 kDa

C	T	M	M+T

C	T	M	M+T

C	T	M	M+T

100 kDa

150 kDa

Supplementary figure 5. Full uncropped Blots image(s) for SOD (20 kDa)

10 kDa

15 kDa

20 kDa

25 kDa

37 kDa

50 kDa

100 kDa

75 kDa

150 kDa

250 kDa

Supplementary figure 5. Full uncropped Blots image(s) for NF-κB (130 kDa)

C	T	M	M+T

C	T	M	M+T

C	T	M	M+T

Supplementary figure 6. Full uncropped Blots image(s) for NF-κB p50 (45 kDa)

15 kDa

25 kDa

20 kDa

37 kDa

50 kDa

75 kDa

100 kDa

150 kDa

250 kDa

C	T	M	M+T

C	T	M	M+T

C	T	M	M+T

C	T	M	M+T

C	T	M	M+T

C	T	M	M+T

250 kDa

150 kDa

15 kDa

25 kDa

20 kDa

37 kDa

50 kDa

75 kDa

100 kDa

Supplementary figure 7. Full uncropped Blots image(s) for NF-κB p65 (65 kDa)

C	T	M	M+T

C	T	M	M+T

C	T	M	M+T

10 kDa

15 kDa

20 kDa

25 kDa

37 kDa

50 kDa

75 kDa

100 kDa

250 kDa

150 kDa

Supplementary figure 8. Full uncropped Blots image(s) for β-actin (40-42 kDa)
